# Report of the King's Fund Convalescent Homes Committee

**Published:** 1914-12-26

**Authors:** 


### Report of the King's Fund Convalescent Homes Committee.

### ADOPTED AT THE MEETING OF GOVERNORS AND GENERAL COUNCIL.

(1) The Governors and General Council have this year fixed the sum available for distribution amongst convalescent homes and consumption sanatoria at £6,500.

(2) The number of applications eligible for consideration amounted to forty-eight from convalescent homes and ten from consumption sanatoria, as against forty-six and ten respectively last year.

(3) In view of the present emergency and the increased difficulty in providing for ordinary maintenance which those responsible for the management of charitable institutions have reason to anticipate, the committee have not felt themselves justified in recommending any capital grants to new schemes, or in taking advantage of various offers which would have enabled them to secure a larger number of beds at consumption sanatoria for the use of patients from London hospitals. In dealing with all applications, whether from sanatoria or from convalescent homes, they have thought it more necessary this year than ever to take into consideration the question of comparative urgency of need, as shown by the accounts and balance sheets of the various institutions to December 31, 1913. In the case of consumption sanatoria they have also taken note of the proportion of the total work of each institution which is covered by receipts under the National Insurance Act.

It must not be assumed, therefore, that a reduction in the grant to any particular institution, whether for maintenance or for capital purposes, or even the absence of a grant, implies dissatisfaction.

(4) In dealing with applications from convalescent homes the committee have adhered to their original policy of giving special consideration to the convalescent homes closely connected with London hospitals.

(5) The accommodation secured this year at consumption sanatoria for the use of patients from London hospitals consists, as last year, of forty-four beds at five sanatoria. These are situated as follows:—

Two beds for children at the Children's Sanatorium, Holt, Norfolk; ten beds at the Devon and Cornwall Sanatorium, South Brent, Devon; ten beds at the Kelling Sanatorium, Holt, Norfolk; ten beds at the sanatorium of the National Association for the Establishment and Maintenance of Sanatoria for Workers, Benenden, Kent; twelve beds at the Northampton Sanatorium, Creaton, Northants.

(6) In allocating these beds to hospitals in London the committee have followed the same principle as that adopted last year—viz., that preference should be given to the two consumption hospitals which have at present no country branches, and that the rest of the beds should be divided among the largest of the general hospitals which apply to the Fund.

They accordingly recommend the following allocation:—

Eighteen beds to the City of London Hospital for Diseases of the Chest; nine beds to the Royal Hospital for Diseases of the Chest; six beds to the London Hospital; six beds to Guy's Hospital; five beds to the Middlesex Hospital.

(7) All the sanatoria applying for grants have been inspected on behalf of the Fund, and the committee desire to express their indebtedness to the visitors who undertook this work, which in many cases has necessitated long journeys from London.

### GRANTS RECOMMENDED BY THE CONVALESCENT HOMES COMMITTEE.

### A.—CONSUMPTION SANATORIA.

*(See second part of paragraph 3 of Report.)*

Children's Sanatorium, Holt, Norfolk, £125 in consideration of the reservation of two children's beds during 1915 for the use of certain London hospitals (see paragraphs 5 and 6 of Report).

Daneswood Sanatorium (Jewish), Woburn Sands. Devon and Cornwall Sanatorium, Didworthy, South Brent, £875 in consideration of the reservation of ten beds during 1915 for the use of certain London hospitals (see paragraphs 5 and 6 of Report).

Kelling Sanatorium, Holt, Norfolk, £875 in consideration of the reservation of ten beds during 1915 for the use of certain London hospitals (see paragraphs 5 and 6 of Report).

Mount Vernon Hospital, Northwood, £50.

National Association for the Establishment and Maintenance of Sanatoria for Workers, Benenden, Kent, £875 in consideration of the reservation of ten beds during 1915 for the use of certain London hospitals (see paragraphs 5 and 6 of Report). Northampton Sanatorium, Creaton, Northants, £1,050; in consideration of the reservation of twelve beds during 1915 for the use of certain London hospitals (see paragraphs 5 and 6 of Report).

Royal National Hospital for Consumption, Ventnor.

Total to Consumption Sanatoria ... £3,850.

### B.—CONVALESCENT HOMES.

Alexandra Hospital for Children, Painswick, £25.

Baldwin Brown Home for Convalescent Poor, Herne Bay, £25. Beau Site Convalescent Home, Hastings, £25. Brentwood Convalescent Home for London Children, Brentwood, £50. Bushey and Bushey Heath (Children), Bushey Heath, £25.

Charing Cross Hospital, Limpsfield, £150 with a view to encouraging the admission without payment of patients direct from the hospital. Chelsea Hospital for Women, St. Leonards, £50 with a view to encouraging the admission without payment of patients direct from the hospital. Children's Cottage Hospital, Cold Ash, Newbury, £25 in consideration of convalescent cases. Children's Home Hospital, Barnet, £25 in consideration of convalescent cases.

Convalescent Home for Poor Children, St. Leonards, £25.

Convalescent Police Seaside Home, Hove, £25.

Deptford Medical Mission Convalescent Home, Bexhill, £25.

East London Hospital for Children, Bognor, £75.

Friendly Societies' Convalescent Homes, Dover and Herne Bay, £50.

Great Northern Central Hospital, Clacton-on-Sea, £150 of which £25 to reduce debt.

Highgate Convalescent Home for Children (formerly All Saints' Home), Highgate, £25. Home and Infirmary for Sick Children, Sydenham, Broadstairs, £50. Hospital for Sick Children, Great Ormond Street, Highgate, £100.

King's College Hospital, Hemel Hempstead, £75.

London Hospital Convalescent Home, Tankerton, £100.

Metropolitan Convalescent Institution (Children), Broadstairs, £100. Metropolitan Convalescent Institution, Walton, £100. Middlesex Hospital, Clacton-on-Sea, £350. Mrs. Kitto's Free Convalescent Home, Reigate, £75.

National Hospital for the Paralysed, Finchley, £100 with a view to facilitating the admission of patients without payment.

Paddington Green Children's Hospital, Slough, £100. Poplar Hospital, Walton-on-Naze, £50.

Queen Charlotte's Lying-in Hospital, Kilburn, £50. Queen's Hospital for Children, Bexhill, £150 of which £50 to reduce debt.

St. Andrew's Convalescent Home, Folkestone, £50. St. Andrew's Convalescent Hospital, Clewer, £25. St. Mary's Home for Children, Broadstairs, £75 in consideration of convalescent cases. St. Michael's Home for Men and Women, Westgate-on-Sea, £25. St. Peter's Orphan and Convalescent Homes, Broadstairs, £25 in consideration of convalescent cases. Sunday School Union Convalescent Home, Bournemouth, £25. Sunshine Home of Recovery, Hurstpierpoint, £25. Surgical Home for Boys, Banstead, £25 in consideration of convalescent cases.

Tilford Convalescent Home for Children, Farnham, £25

Victoria Hospital for Children, Chelsea, Broadstairs, £125. Victoria Hospital for Children, Chelsea, Biggin Hill, Kent, £25.

Winifred House Invalid Children's Convalescent Hospital Home, Tollington Park, £25.

Total to Convalescent Homes ... ... £2,650

**Total Grants Recommended to Convalescent Homes and Consumption Sanatoria for the Year 1914 ... ... ... ... £6,500**

